# Age differences in demographic, social and health-related factors associated with loneliness across the adult life span (19–65 years): a cross-sectional study in the Netherlands

**DOI:** 10.1186/s12889-020-09208-0

**Published:** 2020-08-06

**Authors:** Thanée Franssen, Mandy Stijnen, Femke Hamers, Francine Schneider

**Affiliations:** 1 Department of Family Medicine, Care and Public Health Research Institute (CAPHRI), Faculty of Health, Medicine and Life Sciences, Maastricht University, Postbus 616, Maastricht, 6200 MD The Netherlands; 2 Department of Health Services of Research, Care and Public Health Research Institute (CAPHRI), Faculty of Health, Medicine and Life Sciences, Maastricht University, Postbus 616, Maastricht, 6200 MD The Netherlands; 3 Public Health Service South-Limburg, Academic Collaborative Centre for Public Health, Postbus 33, Heerlen, 6400 AA The Netherlands; 4 Public Health Service, Postbus 1150, Limburg-North, Venlo, 5900 BD The Netherlands; 5 Department of Health Promotion, Care and Public Health Research Institute (CAPHRI), Faculty of Health, Medicine and Life Sciences, Maastricht University, Postbus 616, Maastricht, 6200 MD The Netherlands

**Keywords:** Loneliness, Adults, Demographic, social and health-related factors, Age-normative life-stage perspective, Cross-sectional design

## Abstract

**Background:**

Recognition of loneliness as a health concern among adults stresses the need to understand the factors associated with loneliness. Research into factors of influence in the various phases of the adult life span (19–65 years) is scarce. Therefore, the associations between demographic, social and health-related factors and loneliness among young (19–34 years), early middle-aged (35–49 years) and late middle-aged adults (50–65 years) were explored.

**Methods:**

A secondary analysis with a large cross-sectional dataset was performed. Data was collected from September to December 2016 in the Netherlands, by a self-report survey. Loneliness was measured using the De Jong-Gierveld Loneliness Scale. In total, 26,342 adults (19–65 years) participated (response rate: 34%). Multiple logistic regression analyses were performed to examine associations between demographic, social and health-related factors as independent variables, and loneliness as dependent variable among the three age groups.

**Results:**

Prevalence of loneliness among young, early and late middle-aged adults was 39.7, 43.3 and 48.2%, respectively. Living alone, frequency of neighbour contact, perceived social exclusion, psychological distress, psychological and emotional wellbeing were consistently associated with loneliness across the groups. The association between ethnicity and loneliness was stronger among young and early middle-aged adults, compared to late middle-aged adults. Young adults showed the strongest association between contact frequency with friends and loneliness. The strength of association between financial imbalance and loneliness gradually decreased from young to late middle-aged adults. Educational level was associated with loneliness among young adults only, while an association between employment status and loneliness was found solely among early middle-aged adults. For late middle-aged adults only, perceived health was associated with loneliness. Frequency of family contact was associated with loneliness, only among early and late middle-aged adults.

**Conclusion:**

This study indicates that factors associated with loneliness across the adult life span may be understood from an age-normative life-stage perspective. Accordingly, there is no one-size-fits-all approach to reduce loneliness among adults, suggesting that a variety of interventions or an indirect approach may be necessary. Future research should focus on causal relations between factors and loneliness in different age groups, using a longitudinal research-design with, preferably, an even broader set of factors.

## Background

Although loneliness is often stereotyped as a problem specific for the elderly, it is a serious problem for younger adults as well [1]. The prevalence of loneliness among European adults ranges from 7.9% [2] to 45% [3], depending on the measure of loneliness and the country of origin [4]. Many different definitions of loneliness exist. A widely used definition presented by Perlman and Peplau [5] describes loneliness as “the unpleasant experience that occurs when a person’s network of social relations is deficient in some important way, either quantitatively or qualitatively”. This definition, like many others, incorporates three common aspects of loneliness: first, loneliness occurs when there is a discrepancy in the person’s social relationships; second, it is a subjective experience and third, loneliness is a stressful and unpleasant feeling [6].

In addition to loneliness being stressful and unpleasant, it has a significant impact on physical and psychological health as well. Feelings of loneliness are associated with, among others, diabetes [7], cardiovascular diseases [8], sleep disorders [9] and Alzheimer’s disease [10]. It is noteworthy that the mortality excess associated with loneliness is equivalent to other established risk factors such as obesity and substance abuse [11]. The consequences of loneliness are not only significant in terms of health, but also in terms of public health care costs: research shows that loneliness is associated with increased primary care physician visits [12, 13]. A recent review shows that loneliness is likely to be associated with excess health care costs [14]. Moreover, a study in the United Kingdom estimated that the increase in health services usage due to either loneliness or the consequences of loneliness will result in a cost of £12,000 per person over 15 years [15].

Due to the detrimental effects of loneliness for the individual as well as society, loneliness is recognized as a major public health concern [16]. This stimulated the urge to identify factors associated with loneliness. The most important identified demographic factors associated with loneliness include being unmarried, having a low academic degree, and financial problems [17, 18]. Gender is also associated with loneliness, although the results of studies are inconsistent. Some studies report that females are lonelier than males [19, 20], whereas others show that loneliness is more prevalent among males [13, 21]. However, a recent meta-analysis found no gender differences in loneliness among adults [22] In addition to demographic factors, several social factors have been associated with loneliness including living alone [16], having a smaller social network [23], lacking social support and low frequency of social contacts, e.g. seeing friends [24]. On top of that, several physical and psychological health-related factors have been disclosed that increase the likelihood of experiencing loneliness, including, among others, physical functioning impairment, chronic diseases, depression and well-being [18, 25, 26].

Studies have shown that factors associated with loneliness correlate with age [24, 27, 28]. Therefore, it is suggested that loneliness can be understood from a lifespan perspective [24]. This perspective holds that each period in life is characterized by specific behaviours and goals, such as completing school and leaving the parental home for young adults [29]. Whether an individual perceives loneliness or not, depends on the individual’s ability to perform and/or meet these age-normative behaviours and goals. For example, a cross-sectional study showed not having a full-time job is associated with loneliness particular among adults, aged 30–65 years [24]. It is suggested that having a full-time job is the norm for this age-category [24]. Thus, the distribution of factors associated with loneliness diverges across the various life phases. Furthermore, the impact of these factors varies across the different phases of life. Some factors may have a greater impact on loneliness in youth than in elderly, and vice versa. For example, it has been shown that a poor health status has a greater impact on loneliness among youth. A poor health among young adults may lead to them not feeling understood by their peers which places them at risk for loneliness [30, 31], while elderly may anticipate health problems [27].

Although it is acknowledged that factors associated with loneliness vary across the life span, most research focusses on adolescents or older adults (aged 65 years and over). Studies that do focus on younger adults (19–65 years) tend to include broad age groups [24, 27, 32–34]. There is a lack of research that exclusively focusses on adults aged 19–65 years and examines factors associated with loneliness in more narrow age groups [24, 27]. The identification and understanding of these factors is necessary to recognize adults in different phases of life who suffer from loneliness. These insights can be used to advance knowledge in the development of interventions as current interventions mostly fail to reduce loneliness [27, 35]. Therefore, the aim of current study is to explore whether the factors associated with loneliness as previously identified by research vary across the different life phases of the adult life span (19–65 years).

## Method

### Study design

A secondary analysis was performed by using individual-level data of the Adult Health Monitor Limburg 2016. The Adult Health Monitor is a large cross-sectional population-based health survey carried out once every 4 years by all Public Health Services in the Netherlands from September to December [36]. This survey was developed by the Public Health Services in collaboration with the National Institute for Public Health and the Environment (RIVM) and Statistics Netherlands (CBS). It aims to monitor self-reported health of the population aged 19–65 years by means of close-ended question addressing physical/psychological health, well-being, social functioning, lifestyle, participation in society, formal and informal care, child rearing and environmental nuisances. The information on population health status and related topics is used for regional and local policy-making purposes [36].

### Participants

Inclusion criteria for participating in the survey were: (a) living in one of the 33 municipalities of Limburg; (b) aged 19–65 years old on September 1st, 2016; (c) living independently (i.e. not living in an institution for mentally disabled or mental health institution). A total of 76,704 adults aged 19–65 years in Limburg were approached and 26,342 adults responded, resulting in a response rate of 34%. During data preparation, an additional 23 participants were excluded since their questionnaires had too many missing values. The survey protocol stated that a participant should be excluded if missing answers were present for 11 basic questions; birthyear, gender, height, weight, perceived health status, smoking, alcohol consumption, smoking, employment, education, income and giving informal care. Hence, the total sample size comprised 26,319 adults.

### Adult health monitor Limburg procedure

Statistics Netherlands selected participants for the Adult Health Monitor using a stratified random sample design, based on data from the Personal Records Database of the municipalities in Limburg. All 33 municipalities in Limburg were sampled to ensure representativeness for the region. Selected individuals were invited by postal mail to participate in the survey. They could choose to fill-in the questionnaire on paper or to complete the questionnaire online, using a personal login-code. Two reminders (referring individuals to either the postal questionnaire or the online questionnaire) were sent to non-responders using a three-week interval to allow them sufficient time to receive, respond and return the questionnaire.

### Measures

To examine the association between demographic, social and health-related factors and loneliness across different phases of the adult life span, three age groups were created. Therefore, date of birth of each participant was recoded into three categories: 19–34 years being young adults; 35–49 years being early middle-aged adults; 50–65 years being late middle-aged adults. These age cut-off points have been chosen so they represent the life phases of the adult life span [37, 38]. Moreover, the three age categories followed the commonly used age categories for reporting results of the Adult Health Monitor in the Netherlands.

#### Independent factors

Independent factors were categorized in three sets, including demographic factors, social factors and health-related factors.

##### Demographic factors

A series of single-item questions with predetermined response options were used to assess demographic characteristics, including: *gender* (1 = male; 2 = female); *education* (1 = low, 2 = intermediate, 3 = high); and *marital status* (1 = married, living together; 2 = never been married; 3 = divorced; 4 = widowed). The response options for the item assessing *ethnicity* (based on country of origin) were recoded into two categories: Dutch origin (1) and non-Dutch (western and non-western) origin (2). *Financial imbalance* was determined by asking about difficulties to make ends meet and answers were coded as not having difficulties (0 = no difficulties at all; no, but I need to be careful with my expenditures) or having difficulties (1 = yes, I have some difficulties; yes I have many difficulties). Information on *employment status* was gathered with a range of detailed answers categories and were recoded into two categories: not having a paid job (0 = (early) retired, unemployed/job seeker, incapacitated for work, receiving a social assistance benefit, housewife/houseman or student) and having a paid job for at least 1 h a week (1 = a paid job for > 32-h, paid job for 20–32 h, paid job for 12–20 h, paid job for 1–12 h a week).

##### Social factors

The social context was captured by measuring *living arrangement, volunteer work, frequency of contact with family, friends* or *neighbours* and *social exclusion*. Living arrangement was assessed by following open-ended question: “how many people currently live with you in your household?”. This variable was recoded as dichotomous variable: living alone (0) and two-or-more-person household (1). Volunteer work was assessed by the item: “do you do voluntary work?” (no = 1; yes = 2). Information on social contacts was gathered by asking about frequency of telephone, email or written contact or face-to-face meetings and independent responses were required for family members, friends/acquaintances and neighbours. Answer options were recoded as more than twice a month (0 = at least once a week; three times a month; twice a month) and less than twice a month (1 = once a month; less than once a month; seldom/never). Social exclusion was assessed by the item: “do you feel excluded from society”, and answers were recoded as (very) often (1 = often; very often) and seldom, never (2 = sometimes, seldom, never).

##### Health-related factors

Health-related factors were measured through a variety of items and measurement scales. *Limitations in daily activities* was assessed by asking if participants were restricted in performing daily activities, for 6 months or longer, due to health problems. Response options were recoded as not restricted (0 = no, not restricted at all) and restricted (1 = severe restricted; restricted but not sever). *General health* was assessed by the item: “how is your health in general?”, response options were coded as poor (0 = very poor; poor; moderate) and good (1 = good, very good). By asking if participants had a health condition lasting more than 6 months, *chronic diseases* were assessed (response option: no = 0; yes = 1). Information on *informal caregiving* was gathered by asking if the participant provided informal care (yes/no), how many hours a week (open-ended question), since when (less than 3 months; 3 months or more) and if they perceived burden (ranging no burden to overburden). The responses were combined and recoded as no burden (1 = yes, I provide informal care, for 8 h a week or lasting more than 3 months and perceived no burden at all, or slightly burden), overburden (2 = yes, I provide informal care for 8 h a week or lasting more than 3 months and perceive severe, very severe burden or overburden) or not an informal caregiver (8 = yes, I provide informal caregiver, but less than 8 h a week or less than 3 months; no, I do not provide informal care;). A Dutch version of the Kessler Psychological Distress Scale (k10) was used for estimating risk of *depression or anxiety disorders* [39]. The Dutch k10 showed good reliability and validity [40]. Participants rated 10 symptoms on a 5-point Likert scale (1 = never; 5 = always). Total scores were calculated and the variable was operationalized as a dichotomous variable: 0 = no, low or moderate risk (scores 10 to 24), and 1 = high risk (scores 25–50). Finally, positive mental health was assessed by the Dutch Mental Health Continuum-Short Form (MHC-SF [41];). The Dutch MHC-SF has been shown to be reliable and valid [42]. The scale consisted of three subscales: *psychological well-being*, i.e., the ability to function in an optimal way (6 items); *emotional well-being*, i.e., life satisfaction (3 items) and *social well-being*, i.e., participating optimally in society (5 items). The items were answered on a 6-point scale, ranging from never (0) to every day (5). Mean scores were computed separately for each subscale (range score 1–5, high score = high well-being).

#### Outcome measure

The 11-item De Jong-Gierveld Loneliness Scale [43] was used to measure loneliness, the outcome measures of the current study. The scale proved to be reliable and valid in previous research [44, 45]. Participants were required to indicate for each item the extent to which the statement applies to their situation. Three answer options were presented, including: yes; more or less; and no, whereas the “more or less” answers were assigned to the value representing lonely. According to the manual, if two or more item scores were missing, loneliness scale scores were neither computed nor included [46]. The total loneliness score was categorised into not lonely (0–2), moderate loneliness (3–8), severe loneliness (9, 10) and very severe loneliness (11). The scale was recoded as a dichotomous variable: no loneliness = 0 (scores 0–2) and moderate/severe loneliness = 1 (scores 3–11) [43]. The cut-off point of 2 was chosen since this is commonly used for reporting loneliness scores of the Health Monitor in the Netherlands [47] and in previous research (e.g. [48, 49]). Moreover, research has shown that age trends in loneliness were not affected by dichotomizing loneliness scores [50].

### Statistical analysis

All statistical analyses were done with IBM SPSS Statistics, Version 21 for Windows. Given that for this study a very large sample with a stratified sampling design was used, the complex sample procedure was used for statistical analyses, to make valid inferences from the data (computation of more accurate standard errors and confidence intervals) [51]. Weights were calculated by Statistics Netherlands to correct for unequal selection probabilities and selective non-response using data from the Personal Records Database of municipalities about age, gender, marital status, degree of urbanisation, household size, ethnicity, income, and size of geographical units [52]. Since the weights were calculated based on the non-response pattern and national statistics, the weighted sample was representative for the adult population of Limburg in 2016 [52, 53].

Statistical analyses were undertaken by a multistep process. For step 1, descriptive statistics were used to describe the sample. For psychological, emotional and social wellbeing weighted means and standard deviations were reported. For all other independent variables, weighted percentages (WP) and unweighted counts were reported. At step 2, bivariate analysis was performed by simple binary logistic regression to assess the unadjusted association between each of the independent variables and loneliness. The results of the bivariate analyses were presented as crude odds ratios (COR) and their corresponding 95% confidence intervals (95% CI). All factors significant at *p* < 0.05 in the bivariate analysis were included in the next step. During the multivariate analyses in step 3, a multiple logistic regression analysis was performed to assess the adjusted association between loneliness and each set of independent variables (demographic, social, and health-related factors). For each independent variable of the three sets, the adjusted odds ratio (AOR) and its corresponding 95% CI were presented. Finally, in step 4, an overall multiple logistic regression analysis was carried out. The factors significant at *p* < 0.05 of step 3 were entered in the final multiple regression model. For the final regression model, a stepwise backward elimination regression method was used. At each step of the modelling, the association of the factors on loneliness was assessed. Factors significant at *p* < 0.05 were kept for the next step of the modelling. These four steps were repeated for each age category (19–34 years; 35–49 years; 50–65 years).

## Results

### Characteristics of the participants (descriptive statistics, step 1)

A total of 26,319 adults were included in this study, comprising of 6143 young adults (23.3%), 8418 early middle-aged adults (32.0%) and 11,758 late middle-aged adults (44.7%). In all three age groups, the sample was evenly distributed between females and males (Table 1). The majority in each group was of Dutch origin, was employed, perceived no financial imbalance and was living with 2 or more persons together. Most of the young adults had never been married, while most early middle-aged adults and late middle-aged adults were married or living together. In all age groups, most individuals communicated more than twice a month with their family, friends or neighbours. More late middle-aged adults reported their general health as poor, limitations in daily activities and chronic diseases compared to young and early middle-aged adults. In each age group more than two thirds had a moderate or high risk of depression or an anxiety disorder.

**Table 1:** Participants demographic, social and health-related characteristics and loneliness prevalence for each age group

	19–34 years		35–49 years		50–65 years	
	n (%)	Lonely, n (%)	n (%)	Lonely, n (%)	n (%)	Lonely, n (%)
**Demographic factors**						
Gender						
Male	2528 (51.1)	887 (40.6)	3790 (50.0)	1462 (45.4)	5606 (50.1)	2641 (52.0)
Female	3615 (48.9)	1155 (38.9)	4628 (50.0)	1646 (41.2)	6152 (49.9)	2518 (44.4)
Education						
Low	579 (12.6)	351 (63.1)	1431 (21.4)	778 (57.9)	3868 (36.9)	2016 (53.6)
Intermediate	2547 (47.9)	975 (40.4)	3319 (41.1)	1309 (41.7)	4096 (36.7)	1906 (47.7)
High	2297 (39.5)	692 (31.5)	2930 (36.5)	987 (36.1)	3017 (26.3)	1173 (41.1)
Ethnicity						
Dutch origin	5218 (78.7)	1592 (35.1)	6987 (76.9)	2394 (38.4)	10,021 (81.7)	4949 (46.0)
Non-Dutch origin	925 (21.3)	450 (58.2)	1431 (23.1)	714 (60.1)	1737 (18.3)	910 (58.0)
Employment Status						
Unemployed	1140 (24.8)	604 (54.9)	992 (14.9)	627 (70.2)	3402 (33.3)	1892 (57.7)
Employed	4281 (75.2)	1414 (34.7)	6669 (85.1)	2441 (38.4)	7551 (66.7)	3193 (43.3)
Financial imbalance						
No	4486 (81.6)	1441 (33.6)	6305 (80.0)	2203 (37.0)	9056 (80.1)	3766 (42.6)
Yes	915 (18.4)	571 (67.0)	1358 (20.0)	864 (67.3)	1925 (19.9)	1336 (70.7)
Marital status						
Married/living together	3126 (44.6)	881 (33.4)	6681 (77.2)	2188 (38.5)	9246 (76.5)	3673 (42.7)
Never been married	2888 (53.9)	1103 (44.6)	982 (13.4)	527 (62.1)	738 (7.6)	435 (65.6)
Divorced	76 (1.4)	38 (53.1)	679 (8.9)	359 (55.9)	1284 (12.3)	795 (68.4)
Widowed	7 (0.2)	5 (74.4)	45 (0.5)	21 (59.1)	419 (3.6)	223 (57.5)
**Social factors**						
Living arrangement						
Living alone	776 (15.4)	352 (57.0)	895 (12.9)	507 (63.4)	1683 (16.9)	1042 (68.1)
2 or more persons	5324 (84.6)	1668 (36.5)	7431 (87.1)	2556 (39.9)	9820 (83.1)	3982 (43.7)
Volunteer work						
No	4133 (75.8)	1586 (40.9)	5367 (69.8)	2289 (45.7)	8018 (72.6)	3883 (49.8)
Yes	1314 (24.2)	444 (35.8)	2329 (30.2)	801 (37.5)	2994 (27.4)	1237 (43.8)
Informal caregiver						
No	5041 (93.8)	1879 (39.5)	6601 (86.5)	2630 (43.1)	8053 (75.0)	3860 (49.6)
Yes	383 (6.2)	140 (41.2)	1061 (13.5)	440 (43.0)	2817 (25.0)	1185 (43.2)
Family contact						
More than twice a month	5198 (94.5)	1871 (38.4)	7241 (93.5)	2678 (41.1)	10,062 (91.0)	4423 (45.3)
Less than twice a month	246 (5.5)	159 (63.6)	451 (6.5)	324 (76.5)	954 (9.0)	694 (76.9)
Friends contact						
More than twice a month	5139 (94.4)	1784 (37.2)	6800 (88.0)	2394 (38.4)	9382 (85.1)	3845 (42.5)
Less than twice a month	304 (5.6)	245 (82.7)	895 (12.0)	701 (78.6)	1630 (14.9)	1272 (80.0)
Neighbours contact						
More than twice a month	3816 (67.4)	1183 (33.1)	104 (78.5)	2127 (37.6)	8785 (79.0)	3640 (42.6)
Less than twice a month	1626 (32.6)	846 (53.4)	1583 (21.5)	958 (63.8)	2204 (21.0)	1461 (68.6)
Societal exclusion						
(Very) Often	545 (10.1)	1613 (34.8)	665 (8.9)	2553 (39.0)	1029 (9.5)	4949 (43.7)
Sometimes, seldom, never	5264 (89.9)	424 (85.1)	7413 (91.1)	542 (87.0)	10,375 (90.5)	877 (90.6)
**Health-related factors**						
General health						
Poor	797 (13.0)	442 (64.2)	1593 (20.3)	935 (68.2)	3580 (31.7)	2201 (38.9)
Good	5339 (87.0)	1597 (36.2)	6805 (79.7)	2166 (37.1)	8134 (68.3)	2940 (68.5)
Limitations in daily activities						
No	4894 (81.5)	1418 (35.5)	6127 (74,0)	1969 (37.2)	6916 (58.7)	2535 (39.6)
Yes	1131 (18,5)	587 (58.4)	2136 (26,0)	1080 (60.6)	4572 (41.3)	2518 (60.7)
Psychological Distress						
No/low risk	3522 (57.5)	715 (24.8)	5068 (60,1)	1281 (29,8)	6735 (57.2)	2101 (33.4)
Moderate/high risk	2539 (42.5)	1327 (60.6)	3246 (39,9)	1809 (64.0)	4899 (42.8)	3029 (68.0)
Caregiver’s burden						
No burden	337 (5.5)	109 (37.0)	902 (11.3)	345 (394)	2426 (21.7)	946 (40.3)
(Extreme) high/overburden	46 (0.7)	31 (74.1)	159 (2.2)	95 (61.8)	391 (3.3)	239 (62.5)
Chronic disease						
No	4941 (81.5)	1500 (36.7)	6018 (71.9)	1997 (39.2)	6665 (56.5)	2489 (40.3)
Yes	1188 (18.5)	538 (53.2)	2374 (28.1)	1103 (53.7)	5029 (43.5)	2641 (58.3)
Psychological wellbeing (M + S.D.)	3.69 ± 1.0	3.19 ± 1.1	3.62 ± 1.1	3.18 ± 1.1	3.55 ± 1.1	3.10 ± 1.2
Emotional wellbeing (M + S.D.)	3.87 ± 1.0	3.36 ± 1.1	3.84 ± 1.0	3.40 ± 1.1	3.81 ± 1.1	3.34 ± 1.2
Social wellbeing (M + S.D.)	2.82 ± 1.2	2.35 ± 1.1	2.79 ± 1.2	2.39 ± 1.1	2.79 ± 1.2	2.36 ± 1.2

*n* Total number, *M* Mean, *S.D.* Standard deviation

Overall, 10,309 (WP: 44.3) individuals experienced loneliness. Among young adults, 2042 individuals reported feelings of loneliness (WP: 39.7). Of those categorized as early-middle aged adults, 3108 individuals experienced loneliness (WP: 43.3%) and 5159 late middle-aged adults experienced loneliness (WP: 48.2).

### Associations between each factor and loneliness (bivariate analysis, step 2)

In each age group, all factors were significantly associated with loneliness, except gender; this factor was significantly negatively associated with loneliness only for early and late middle-aged adults (Table S1 – See additional file 1). Across all three age groups, the strongest association with loneliness was found for those who often felt excluded from society.

### Associations between factors within a set and loneliness (multivariate analysis, step 3)

All demographic factors entered into the multivariate model were significantly associated with loneliness for all three age groups (Table S1 – See additional file 1). Moreover, all social factors, except volunteer work, were significantly associated with loneliness for all three age groups; volunteer work was only for early middle-aged adults significantly negatively associated with loneliness. Finally, regarding health-related factors, overburdening of informal caregivers was significantly positively associated with loneliness only among young adults. Besides, general health was significantly negatively associated with loneliness only among early and late middle-aged adults.

### Final factors associated with loneliness (multivariate analysis, step 4)

The final overall model identified several factors as significantly associated with loneliness across all three age groups and, besides, the magnitudes of these associations were nearly similar across the age groups (Table 2). These factors included living arrangement, frequency of contact with neighbours, social exclusion, psychological distress, and emotional and psychological wellbeing. Moreover, some factors were significantly positively associated with loneliness for all three age groups, but the magnitudes of the associations varied across the three age groups. Those factors included ethnicity, perceived financial imbalance and less than twice a month contact with friends.

**Table 2:** Overall significant adjusted odds ratio for loneliness for each age group

	19–34 years	35–49 years	50–65 years
	Model pseudo *R*^2^ 0.410	Model pseudo *R*^2^ 0.364	Model pseudo *R*^2^ 0.378
	AOR (95% CI)	AOR (95% CI)	AOR (95% CI)
**Demographic factors**			
Gender			
Male^a^			
Female		0.76 (0.65–0.88)	0.73 (0.65–0.83)
Education			
Low^a^			
Intermediate	0.65 (0.47–0.90)		
High	0.53 (0.38–0.73)		
Ethnicity			
Dutch Origin^a^			
Non-Dutch Origin	2.06 (1.60–2.65)	2.16 (1.78–2.62)	1.27 (1.08–1.49)
Employment Status			
Not currently employed^a^			
Employed		0.71 (0.56–0.90)	
Financial imbalance			
No^a^			
Yes	2.00 (1.56–2.56)	1.54 (1.25–1.91)	1.30 (1.10–1.54)
Marital status			
Married/living together^a^			
Never been married			1.47 (1.10–1.97)
Divorced			1.48 (1.14–1.93)
Widowed			1.37 (0.96–1.96)
**Social factors**			
Living arrangement			
Living alone^a^			
2 or more persons	0.57 (0.43–0.75)	0.58 (0.45–0.75)	0.69 (0.53–0.89)
Volunteer work			
No^a^			
Yes			
Family contact			
More than twice a month^a^			
Less than twice a month		1.91 (1.30–2.80)	2.03 (1.58–2.61)
Friends contact			
More than twice a month^a^			
Less than twice a month	5.93 (3.82–9.22)	3.34 (2.55–4.37)	3.237 (2.65–3.96)
Neighbours contact			
More than twice a month^a^			
Less than twice a month	1.55 (1.27–1.90)	1.63 (1.35–1.98)	1.631 (1.39–1.91)
Society exclusion			
(Very) often^a^			
Sometimes, never	0.34 (0.22–0.49)	0.41 (0.30–0.57)	0.41 (0.30–0.56)
**Health-related factors**			
General health			
Poor			
Good			0.78 (0.67–0.90)
Limitations in daily activities			
No^a^			
Yes			
Psychological Distress			
No/low risk^a^			
Moderate/high risk	2.15 (1.77–2.63)	1.908 (1.61–2.26)	1.76 (1.54–2.01)
Caregiver’s burden			
Not an informal caregiver^a^			
No burden			
Overburden			
Presence of chronic disease			
No^a^			
Yes			
Psychological wellbeing (one unit increase)	0.67 (0.58–0.77)	0.69 (0.62–0.77)	0.70 (0.64–0.76)
Emotional wellbeing (one unit increase)	0.61 (0.53–0.71)	0.68 (0.60–0.76)	0.61 (0.55–0.67)
Social wellbeing (one unit increase)			

Only factors significant at *P* < 0.05 in the final model are presented

*AOR* Adjusted odds ratios, *CI* Confidence interval

^a^ reference category

Moreover, some factors were significantly associated with loneliness, but those factors were not consistent for all three age groups. Being female and having an intermediate or high education level were significantly associated with loneliness, but only among young adults. Only for early middle-aged adults, being employed was significantly associated loneliness. Furthermore, having more than twice a month contact with family was significantly associated with loneliness solely for early and late middle-aged adults. In terms of health, a good health was significantly associated with loneliness only among late middle-aged adults.

## Discussion

The recognition of loneliness as a major health issue for adults [1] emphasizes the need to gain insight into the nature of loneliness among adults. Current research includes broad age groups and therefore provides insufficient insight into the factors of influence in the various phases of the adult life span (19–65 years). To fill in this gap, the present study explored if the associations between demographic, social and health-related factors and loneliness varied for young adults (19–34 years), early middle-aged (35–49 years) and late middle-aged adults (50–65 years) using a large dataset.

Results of the present study showed that some factors were associated with loneliness in each age group. In other words, those factors are consistently associated with loneliness throughout the adult life span. All these universal factors, with the exception of living arrangement and contact with neighbours, may be indicators of psychological health. Surprisingly, more psychological indicators were associated with loneliness compared to physical health indicators across all three age groups. The association between psychological health and loneliness in different age groups has been observed in a variety of other studies [54]. The current study builds upon this existing literature by showing that there is an association between psychological factors and loneliness among adults irrespective of life stage. This is in line with prior research showing no age-related differences in the association between depression and loneliness [55, 56]. However, this raises the question why psychological health is associated with loneliness regardless the life stage. A plausible explanation is that individuals perceiving psychological problems are among the most excluded individuals in society [57, 58], which, in return, may induce feelings of loneliness [59, 60].

Although some factors appeared to be universal, results clearly showed that certain factors associated with loneliness varied across the age groups. We therefore believe that loneliness may be understood from an age-normative life-stage perspective. This is supported by the results of the current study in two important ways. Firstly, some factors associated with loneliness were present in specific age groups only. Education emerges as an age-specific factor, since it was negatively associated with loneliness among young adults only. This is in line with previous research [33]. Education is more normative for young adults and they are more likely to be expected to strive for educational goals as compared to early and late middle-aged adults [31]. Likewise, employment status appeared as an age-specific factor. Having a job was negatively associated with loneliness among solely early middle-aged adults, as reported in previous research [24]. During this life phase it is more normative to work and adults perceive more financial obligations regarding family compared to the young and late middle-aged life phases [61]. Frequency of family contact was also an age-specific factor. As also reported in previous research [62], this factor was positively associated with loneliness among both early and late middle-aged adults. The importance of family increases with age. Early and late middle-aged adulthood is often marked with childcare responsibilities, commitments to elderly relatives and it suggested that individuals of those ages identify themselves through their relationships with family members [63, 64]. Furthermore, marital status was an age-specific factor for late middle-aged adults. This finding is consistent with previous research [27]. According to the socioemotional selectivity theory [65], importance of emotional closeness increases with age. Hence, not being in a romantic relationship is particularly important in relation to loneliness among late middle-aged adults. We also found that perceived health was associated with loneliness among late middle-aged adults only and thus appeared to be an age-specific factor. This is in contrast with previous research showing an association between poor general health and loneliness among adults of all ages [27]. As discussed earlier, if an individual perceives life events as non-normative for his or her age, loneliness may manifest.

Since poor health is non-normative for all three age groups, we expected to find this association in all age groups. However, this finding may be explained by the higher prevalence of a poor general health status among late middle-aged adults in this study.

Secondly, the age-normative life-stage perspective towards loneliness is supported because the strength of the association of some factors and loneliness differed across the age groups. Results of the current study showed that the strength of the association between being of non-Dutch origin and loneliness was higher among young and early middle-aged adults than among late middle-aged adults. Loneliness is linked to ethnic identity, and the stability of this identity varies across the life span due to normative life events [66, 67]. Young and early middle-aged adulthood is a period of many normative life events, such as completing school, entering the labour market and becoming parent. This period is linked to greater instability of ethnic identity compared to late middle-aged adults. Nevertheless, the result of the current study is in contrast with previous research [33], where an association between ethnicity and loneliness among adults of all ages was found. Methodological differences in assessing and categorizing ethnicity make a clear comparison difficult. Results of the current study may be influenced by the relatively small size of the non-Dutch population. Furthermore, the strength of the association between financial imbalance and loneliness gradually decreased from young to late middle-aged adulthood. Although no studies have yet examined this association across the different phases of the adult life span (19–65 years), it is suggested that financial imbalance affects loneliness through an age-graded pathway [24]. With increasing age, outdoor leisure activities shift to home-based activities [68]. Since home-based activities are generally less expensive, financial difficulties may affect late adults less compared to young adults. However, this should be investigated in more detail. Lastly, the association between having less frequent contact with friends and loneliness was stronger among young adults compared to early and late middle-aged adulthood, which is in line with previous research [34, 48, 69]. Previous research revealed that young adults are more focused on their friendships as compared to early and late middle-aged adults [70]. Young adults are still discovering their identity, which emphasizes the importance of turning to friends for role socialization and leisure activities [71].

Furthermore, it is important to be aware that loneliness is a multidimensional concept, including emotional and social loneliness [72]. It has been suggested that some factors are associated with emotional loneliness, whereas other factors appeared to be associated with social loneliness. This stresses the importance of further research regarding loneliness among adults.

### Limitations of the study

Several limitations should be addressed. Some essential factors were not included in the current study, since they were not measured by the Adult Health Monitor. For instance, relationship quality has been found as an important factor associated with loneliness [23], but this factor was not measured by the Adult Health Monitor. Furthermore, given the cross-sectional design of this study, no causal relationships among the variables can be inferred. We could therefore not test assumptions based on prior research, such as that psychological distress predicts loneliness [55]. Therefore, a reciprocal relationship between all included factors and loneliness may exist [55]. Besides, some factors may act as a mediator or moderator. It has been shown that gender moderates the association between marital status and loneliness [50]. Examining the reciprocal relationship between various factors and loneliness and moderation/mediation effects was beyond the scope of the current study. This should be considered in future research to get a more thorough understanding of those factors associated with loneliness across different age groups. Moreover, the use of a self-reporting survey may increase the risk of social desirability bias. Individuals tend to bias their responses in an admirable and positive way to sensitive questions, such as questions regarding income, taxes and deviant behavior [73]. As the Adult Health Monitor included several sensitive questions, such as questions regarding financial imbalance and psychological problems, the exact prevalence of individuals suffering from those conditions may be underestimated. Consequently, the association between those factors and loneliness may be weaker than in reality. Finally, the choice of the three age categories is based on the standard guidelines for reporting results of the Adult Health Monitor in the Netherlands and may deviate from age-spans used in other countries, thereby restricting generalization of our findings to other countries.

## Conclusion

This cross-sectional study explored demographic, social and health-related factors associated with loneliness across the adult life span. The present study showed that some factors were consistently associated with loneliness across different life phases, while other factors appeared to be age-specific in the association with loneliness. Although more longitudinal research is needed to explore the causal relations between demographic, social and health-related factors and loneliness, this study underscores the notion that an age-normative life-stage perspective should be adopted regarding factors associated with loneliness during the adult life span. Furthermore, the set of factors associated with loneliness differs across the phases of the adult life span, suggesting that there is no ‘one-size-fits-all’ approach to reduce loneliness. The broad set of factors involved, also supports the suggestion that a broader, indirect approach may alleviate loneliness [49]. Hence, the reduction of loneliness may be a secondary outcome in (existing) initiatives that are not directly intended to impact on loneliness. For example, interventions connecting individuals for other purposes, such as fitness groups to improve health, may be effective to reduce loneliness [74]. In conclusion, it should be acknowledged that the factors associated with loneliness vary across different age groups and therefore policy-makers and intervention developers should take these factors into account in efforts to reduce loneliness among adults.

## Supplementary information

**Additional file 1.** Demographic, social and health-related factors associated with loneliness for the three age groups (crude and adjusted odds ratios). This table shows the results of step 3 of the multivariate analysis. It presents the associations between factors within a set and loneliness.

## Data Availability

The dataset supporting the conclusions of this article is included within the article and its additional file. The dataset analysed during the current study is available upon reasonable request from the second author (M.S.) and third author (F.H.).

## Abbreviations

RIVM: National Institute for Public Health and the Environment
CBS: Statistics Netherlands
K10: 10-item Kessler Psychological Distress Scale
MHC-SF: Mental health Continuum-Short Form
WP: Weighted percentage
CI: Confidence interval
COR: Crude odds ratio
AOR: Adjusted odds ratio
M: Mean
S.D: Standard deviation
n: Number
