# Supplementary material for: Age differences in demographic, social and health-related factors associated with loneliness across the adult life span (19–65 years): a cross-sectional study in the Netherlands
Source: BMC Public Health. 2020 Aug 6;20:1118. doi: 10.1186/s12889-020-09208-0 (PMC7409622; doi:10.1186/s12889-020-09208-0)
Supplement: Supplementary file 1 — Additional file 1. Demographic, social and health-related factors associated with loneliness for the three age groups (crude and adjusted odds ratios). This table shows the results of step 3 of the multivariate analysis. It presents the associations between factors within a set and loneliness. [file 12889_2020_9208_MOESM1_ESM.docx]

Table S1. Demographic, social and health-related factors associated with loneliness for the three age groups (crude and adjusted odds ratios)

|  | **19-34 years** | | | | | **35-49 years** | | | | | | **50-65 years** | | | | |  |
| --- | --- | --- | --- | --- | --- | --- | --- | --- | --- | --- | --- | --- | --- | --- | --- | --- | --- |
|  | COR (95% CI) | | AOR (95% CI) | | | COR (95% CI) | | | AOR (95% CI) | | | COR (95% CI) | | | AOR (95%) | |  |
| **Demographic factors** |  |  | |  | | |  |  | |  |  | |  |  | |  | |
| Gender  Male^a^  Female | 0.93 (0.80-1.08) | |  | | | **0.84** (0.75-0.95) | | | **0.73** (0.64-0.84) | | | **0.74** (0.67-0.81) | | | **0.64** (0.57-0.71) | |  |
| Education  Low^a^  Intermediate  High | 0.395  **0.40** (0.31-0.51)  **0.27** (0.21-0.34) | | **0.55** (0.43-0.71)  **0.47** (0.36-0.60) | | | **0.52** (0.45-0.61)  **0.41** (0.35-0.49) | | | **0.77** (0.64-0.93)  **0.71** (0.59-0.85) | | | **0.78** (0.70-0.88)  **0.60** (0.53-0.69) | | | 0.91 (0.80-1.04)  **0.75** (0.66-0.86) | |  |
| Ethnicity  Dutch origin^a^  Non-Dutch origin | **2.58** (2.09-3.17) | | **2.04** (1.65-2.52) | | | **2.43** (2.07-2.84) | | | **2.07** (1.75-2.45) | | | **1.62** (1.41-1.86) | | | **1.41** (1.22-1.64) | |  |
| Employment Status  Not currently employed^a^  Employed | **0.44** (0.37-0.52) | | **0.72** (0.59-0.87) | | | **0.26** (0.22-0.32) | | | **0.44** (0.36-0.54) | | | **0.56** (0.50-0.62) | | | **0.66** (0.59-0.75) | |  |
| Financial imbalance  No^a^  Yes | **4.02**(3.33-4.86) | | **3.17** (2.60-3.87) | | | **3.51** (2.97-4.14) | | | **2.43** (2.03-2.91) | | | **3.26** (2.83-3.75) | | | **2.40** (2.07-2.80) | |  |
| Marital status  Married/living together^a^  Never been married  Divorced  Widowed | **1.61** (1.38-1.87)  2.26 (0.95-5.40)  5.78 (0.98-34.12) | | **1.43** (1.21-1.68)  1.37 (0.57-3.31)  2.26 (0.21-23.76) | | | **2.62** (2.17-3.16)  **2.03** (1.60-2.57)  **2.31** (1.05-5.10) | | | **2.05** (1.67-2.52)  **1.40** 1.09-1.78)  2.06 (0.84-5.09) | | | 2.56 (0.10-3.11)  **2.90** (2.46-3.42)  **1.81** (1.39-2.36) | | | **2.15** (1.75-2.63)  **2.31** (1.93-2.75)  **1.68** (1.29-2.20) | |  |
| **Social factors** |  |  | |  |  | |  |  | |  |  | |  |  | |  | |
| Living arrangement  Living alone^a^  2 or more persons | **0.43** (0.35-0.54) | | **0.47** (0.37-0.60) | | | **0.38** (0.31-0.47) | | | **0.47** (0.38-0.58) | | | **0.36** (0.32-0.42) | | | **0.41** (0.35-0.47) | |  |
| Volunteer work  No^a^  Yes | **0.81** (0.68-0.96) | | 0.89 (0.74-1.08) | | | **0.71** (0.62-0.82) | | | **0.83** (0.73-0.97) | | | **0.78** (0.70-0.88) | | | 0.92 (0.81-1.04) | |  |
| Family contact  More than twice a month^a^  Less than twice a month | **2.80** (1.93-4.08) | | **2.17** (1.51-3.13) | | | **4.42** (3.30-5.92) | | | **2.53** (1.73-3.70) | | | **4.03** (3.35-4.84) | | | **2.45** (1.97-3.05) | |  |
| Friends contact  More than twice a month^a^  Less than twice a month | **8.08** (5.57-11.71) | | **5.60** (3.82-8.22) | | | **5.89** (4.68-7.40) | | | **3.88** (3.03-4.98) | | | **5.40** (4.59-6.35) | | | **3.76** (3.15-4.50) | |  |

**Table S1. (continued)**

|  | **19-34 years** | | | | | **35-49 years** | | | | | | **50-65 years** | | | | | | |  |
| --- | --- | --- | --- | --- | --- | --- | --- | --- | --- | --- | --- | --- | --- | --- | --- | --- | --- | --- | --- |
|  | COR (95% CI) | | AOR (95% CI) | | | COR (95% CI) | | | AOR (95% CI) | | | COR (95% CI) | | | | AOR (95% CI) | | |  |
| Neighbours contact  More than twice a month^a^  Less than twice a month | **2.31** (1.97-2.72) | | **1.77** (1.48-2.12) | | | **2.92** (2.51-3.40) | | | **1.97** (1.97-2.34) | | | **2.95** (2.59-3.36) | | | | **1.96** (1.70-2.25) | | |  |
| Society exclusion  (very) often^a^  Seldom, never | 0**.09** (0.07-0.13) | | **0.13** (0.09-0.17) | | | **0.10** (0.07-0.13) | | | **0.15** (0.11-0.20) | | | **0.08** (0.07-0.10) | | | | **0.12** (0.09-0.16) | | |  |
| **Health-related factors** |  |  | |  |  | |  |  | |  |  | |  | |  | |  | | |
| General health  Poor^a^  Good | **0.32** (0.25-0.40) | | 0.99 (0.73-1.32) | | | **0.28** (0.24-0.32) | | | **0.67** (0.52-0.86) | | | **0.29** (0.26-0.33) | | | | **0.69** (0.59-0.81) | | |  |
| Limitations in daily activities  No^a^  Yes | **2.57** (2.13-3.19) | | 1.25 (0.96-1.63) | | | **2.60** (2.26-2.99) | | | 1.09 (0.86-1.38) | | | **2.36** (2.13-2.62) | | | | 0.96 (0.82-1.12) | | |  |
| Psychological Distress  No/low risk^a^  Moderate/high risk | **4.66** (3.96-5.48) | | **2.48** (2.04-3.01) | | | **4.20** (3.68-4.79) | | | **1.97** (1.67-2.33) | | | **4.23** (3.82-4.69) | | | | **1.87** (1.64-2.13) | | |  |
| Caregiver’s burden  Not an informal caregiver^a^  No burden  Overburden | 0.90 (0.67-1.21)  **4.38** (2.18-8.79) | | 0.86 (0.62-1.18)  **2.74** (1.24-6.04) | | | 0.86 (0.71-1.04)  **2.13** (1.28-3.55) | | | 0.89 (0.72-1.091)  1.16 (0.62-2.157) | | | **0.68** (0.61-0.77)  **1.69** (1.30-2.21) | | | | **0.84** (0.73-0.96)  1.14 (0.82-1.58) | | |  |
| Chronic disease  No^a^  Yes | **1.96** (1.64-2.35) | | 1.07 (0.83-1.38) | | | **1.80** (1.57-2.05) | | | 0.91 (0.74-1.12) | | | **2.21** (1.88-2.29) | | | | 1.13 (0.98-1.31) | | |  |
| Psychological wellbeing  (one unit increase) | **0.37** (0.36-0.41) | | **0.65** (0.57-0.75) | | | **0.43** (0.40-0.46) | | | **0.69** (0.62-0.78) | | | **0.41** (0.39-0.44) | | | | **0.71** (0.64-0.77) | | |  |
| Emotional wellbeing  (one unit increase) | **0.34** (0.31-0.38) | | **0.60** (0.53-0.69) | | | **0.38** (0.35-0.41) | | | **0.63** (0.56-0.71) | | | **0.34** (0.32-0.37) | | | | **0.57** (0.52-0.62) | | |  |
| Social wellbeing  (one unit increase) | **0.52** (0.48-0.56) | | **0.87** (0.78-0.97) | | | **0.56** (0.52-0.59) | | | **0.91** (0.84-0.10) | | | **0.52** (0.50-0.55) | | | | **0.90** (0.84-0.97) | | |  |
| *Note.* COR = crude odds ratios; AOR = adjusted odds ratios  A = reference category  Significant crude odds ratios (p< 0.05) are presented in bold | | | | | | | | | | | |  | |  | | | |  |  |
